# Cholinesterase Inhibitors from Plants and Their Potential in Alzheimer’s Treatment: Systematic Review

**DOI:** 10.3390/brainsci15020215

**Published:** 2025-02-19

**Authors:** Maryam N. ALNasser, Ghadir M. Alboraiy, Eman M. Alsowig, Fatimah M. Alqattan

**Affiliations:** Department of Biological Sciences, College of Science, King Faisal University, P.O. Box No. 400, Al-Ahsa 31982, Saudi Arabia; 221415450@student.kfu.edu.sa (G.M.A.); 221430233@student.kfu.edu.sa (E.M.A.); 220005106@student.kfu.edu.sa (F.M.A.)

**Keywords:** Alzheimer’s disease, cholinesterase inhibitors, medicinal plants, neurodegenerative diseases, plant-based treatment

## Abstract

Introduction: Alzheimer’s disease (AD) is a neurodegenerative disorder characterized by memory loss and cognitive decline, primarily due to dysfunction of acetylcholine caused by acetylcholinesterase and butyrylcholinesterase. While synthetic cholinesterase inhibitors like donepezil, rivastigmine, and galantamine are commonly used, they have notable side effects, prompting interest in natural alternatives. Medicinal plants, rich in bioactive compounds like flavonoids and alkaloids, have shown potential as cholinesterase inhibitors with additional antioxidants and anti-inflammatory benefits. This study aimed to evaluate the cholinesterase-inhibiting effects of various plant species and their compounds to identify new therapeutic candidates and reduce side effects. Method: A PRISMA-compliant review was conducted, screening studies from multiple databases, with a final inclusion of 64 in vivo studies. Results: These studies highlighted plant extracts such as *Ferula ammoniacum*, *Elaeagnus umbellata*, *Bacopa monnieri*, and *Centella asiatica*, which improved memory, reduced oxidative stress, and provided neuroprotection. Some extracts also reduced amyloid plaques, enhanced neuronal integrity, and restored cholinesterase activity, indicating their potential as therapeutic agents for AD and other neurodegenerative diseases. Conclusions: The findings underscore the promise of plant-based compounds in treating cognitive decline and cholinergic dysfunction in AD, advocating for further research into their therapeutic potential.

## 1. Introduction

**Figure 1:**
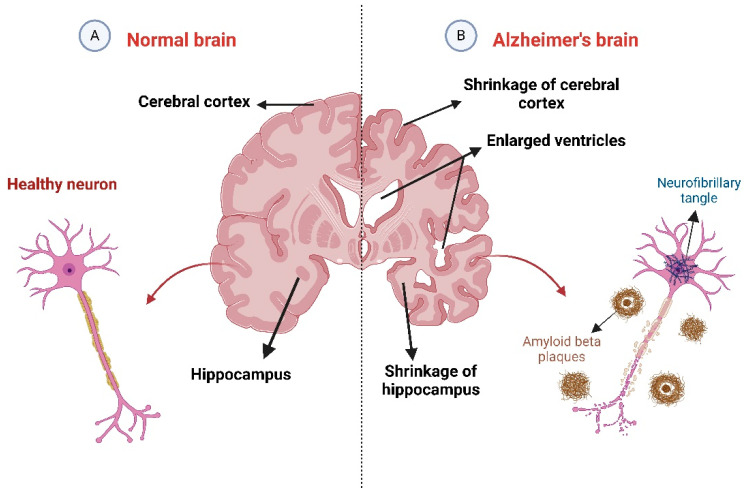
The physiological structure of the brain and neurons in (**A**) healthy brain and (**B**) brain affected by Alzheimer’s disease (AD). The figure was created using BioRender (https://www.biorender.com/).

Dementia refers to cognitive impairments typical for Alzheimer’s disease (AD). AD is characterized by neurodegeneration due to neuronal cell death, starting in the entorhinal cortex of the hippocampus, primarily affecting individuals aged 65 and older [1]. However, it can also affect about 5% of those under 65, termed early-onset Alzheimer’s disease [2,3]. AD affects approximately 50 million people worldwide, and this number is expected to triple by 2050, highlighting its significance as a global health issue. The hallmark features of AD include neurofibrillary tangles and amyloid plaques, which disrupt cholinergic neurons, leading to cognitive decline (Figure 1). Early symptoms include memory problems, sleep issues, and mood disorders, progressing to difficulties in walking and communicating in later stages [4].

The main pathological features include amyloid beta (Aβ) accumulation, tau pathology, and cholinergic dysfunction [4,5]. In AD, cholinergic dysfunction is exacerbated by the altered activity of acetylcholinesterase (AChE) and butyrylcholinesterase (BuChE), enzymes that regulate ACh levels. AChE, responsible for the breakdown of ACh, is typically elevated in AD patients, especially in the cortex and hippocampus, contributing to cognitive impairment. BuChE, although less prominent in healthy brains, increases in AD, particularly in regions affected by amyloid pathology, potentially compensating for the loss of cholinergic neurons [6,7].

Although there is no cure for AD, treatments exist that may slow disease progression or alleviate symptoms. Food and Drug Administration (FDA)-approved medications fall into two categories: those that slow clinical decline and those that relieve symptoms. Some medications, such as aducanumab, a monoclonal antibody approved in 2021, target amyloid plaques to slow cognitive decline. Lecanemab, another monoclonal antibody, was fast-tracked for FDA approval in 2023 for use in mild cognitive impairment or mild dementia. These drugs show promise, but their long-term safety and efficacy are still under evaluation [8,9].

Several drugs focus on symptom management such as cholinesterase inhibitors (ChEIs): rivastigmine, donepezil, galantamine, and tacrine work by increasing ACh levels, improving cognition and memory, though they do not alter the disease’s progression. Donepezil is widely used, while rivastigmine also inhibits BuChE, and galantamine modulates nicotinic receptors [8,9]. Moreover, the N-methyl-D-aspartic acid receptors (NMDARs) antagonist (glutamate inhibitor) memantine regulates glutamate (Glu), preventing neuronal damage from excitotoxicity. It is used for moderate-to-severe AD and can be combined with ChEIs to improve cognitive function [8,9,10].

Current treatments for AD, including cholinesterase inhibitors and memantine, offer limited symptom relief, especially in the early to moderate stages, but do not halt disease progression or address the underlying neurodegeneration. Newer therapies like aducanumab and lecanemab, which target amyloid plaques, may provide benefits in the later stages of the disease. However, these treatments are associated with side effects, ranging from mild symptoms such as nausea, diarrhea, insomnia, muscle cramps, and fatigue to more serious risks like dizziness, headache, hallucinations, heart issues, and brain swelling, particularly with the newer medications [8,9,10]. These challenges underscore the urgent need for novel therapeutic interventions and the development of treatments with fewer side effects to enhance patient outcomes.

Medicinal plants have been utilized for centuries in traditional medicine for their therapeutic properties. These plants are a source of bioactive compounds with potential pharmacological benefits, including antibacterial, anti-inflammatory, and neuroprotective effects [11,12]. Phytochemicals like alkaloids, flavonoids, and terpenoids have demonstrated potential in addressing neurodegenerative diseases (NNDs), including AD, by combating oxidative stress, minimizing inflammation, and protecting neurons [13,14]. Herbal medicines have been explored for their ability to regulate neurotransmitter imbalances, especially acetylcholine, and may serve as potential candidates for AD treatment [15,16,17].

The diversity of plant compounds makes them valuable sources for developing new treatments for AD, offering a holistic approach with fewer side effects compared to synthetic drugs [15,18]. For example, galantamine and rivastigmine (ChEIs), two FDA-approved drugs for AD, are derived from natural sources, highlighting the importance of traditional medicine in modern therapeutic strategies [18,19,20]. Moreover, many plant extracts showed multitarget effects in AD treatment by addressing amyloid plaques, tau aggregation, oxidative stress, inflammation, cholinergic dysfunction, and excitotoxicity, while generally demonstrating safety and low toxicity or no toxicity in studies [18,21,22].

A systematic review was essential to critically assess and synthesize the available evidence on the cholinesterase-inhibiting properties of various plant species, their extracts, and isolated compounds. The review aimed to aggregate current findings, providing a detailed summary of plants with ChEI properties. It also highlighted specific plant species and compounds demonstrating significant cholinesterase inhibition, identifying them as promising candidates for further investigation as potential therapies for AD. Additionally, the review offered insights and recommendations to guide future research, including candidates for clinical trials and deeper exploration of their mechanisms of action, contributing to the development of novel plant-based therapeutic strategies for AD treatment.

## 2. Materials and Methods

This study was performed according to the standards of the Preferred Reporting Items for Systematic Reviews and Meta-Analyses (PRISMA) (Table S1) [23,24]. The study protocol was registered in the International Prospective Register of Systematic Reviews (PROSPERO) under registration number CRD42025585536.

### 2.1. Search Scheme

An electronic literature search was performed from 29 August to 1 September 2024 on Medline (OvidSP), Embase (OvidSP), Web of Science Core Collection, PubMed, and Scopus. The aim was to identify research studies that assess the impact of plant species on cholinesterase enzyme activity. Controlled search vocabularies (MeSH) were used and involved a combination of the following: (a) (in vitro OR neurons) AND (in vivo OR animals), (b) (Plant OR plant products OR Plant extracts OR herb), and (c) (cholinesterase inhibitors OR anti-cholinesterase OR ChEIs OR acetylcholinesterase inhibitors OR anti-acetylcholinesterase OR AChE inhibitor OR anti-AChE OR AChEIs OR butyrylcholinesterase inhibitors OR anti-butyrylcholinesterase OR BChE inhibitor OR anti-BChE OR BChEIs).

### 2.2. Data Extraction Collection and Synthesis

Three independent reviewers (E.A., F.A. and G.A.) participated in data extraction, while the fourth reviewer (M.N.A) assisted in resolving any discrepancies. Relevant data were collected from qualifying publications and organized into tables categorized by study type: in vitro studies involving human or animal neurons, and in vivo animal models. Additionally, the following information was gathered from the relevant studies: authors, publication year, whether the study was conducted in vitro or in vivo, type of enzyme, cell or animal species, scientific name of the plant, type of extracts used, dosage or concentration of the extracts, duration of treatment, route of administration (for in vivo studies), study procedures, overall outcomes, and conclusions. Each eligible study’s data were then qualitatively synthesized in the main text.

A total of (1571) search results were imported into EndNote^®^ 21 and Rayyan^®^ 1.4. 3 softwares for further screening and duplication removal. Subsequently, a manual examination of the title and abstract screening was conducted to identify relevant studies based on the predetermined inclusion criteria. Three independent researchers (M.N.A., E.A., and F.A.) selected studies based on the eligibility criteria for this systematic review.

The review question was the following: Can plants and their components inhibit cholinesterase activity in neurons?

Population (P): in vitro neurons (human or animal), and in vivo animal models of Alzheimer’s.

Intervention (I): Plants and their components.

Comparison (C): Cholinesterase activity in controls (models of Alzheimer’s).

Outcomes (O): Inhibit cholinesterase activity.

### 2.3. Inclusion Criteria

The analysis included all original in vitro studies, encompassing research that utilized established cell lines (e.g., neuronal cell lines) to assess the effects of plant extracts or isolated compounds on cholinesterase (ChE) activity, specifically within the context of Alzheimer’s disease.

Similarly, all in vivo studies involving vertebrate animal models treated with various intervention compounds to induce over-activation of cholinesterase enzymes, as well as Alzheimer’s disease models characterized by impaired cholinergic neurotransmission, were included. These studies must examine the direct effects of plant extracts on cholinesterase enzymes, specifically AChE and/or BChE.

There were no restrictions on the duration, route of administration, or dosage of the plant extracts.

### 2.4. Exclusion Criteria

Studies that assessed plant products influencing enzymes other than ChE were excluded, as were those conducted on isolated AChE and/or BChE enzymes, or in humans, in silico studies, or those employing computer simulations, computational methods, or molecular docking. Additionally, studies involving non-vertebrate animal models or models not related to Alzheimer’s disease were excluded. Studies measuring ChE activity in vivo, but not in the brain (neurons)—such as those analyzing plasma or erythrocyte acetylcholinesterase—were also excluded if they reported no effect on ChE or an increase in its activity. Furthermore, reviews, meta-analyses, case reports, editorials, conference abstracts, and studies lacking control groups were not considered.

## 3. Results

**Figure 2:**
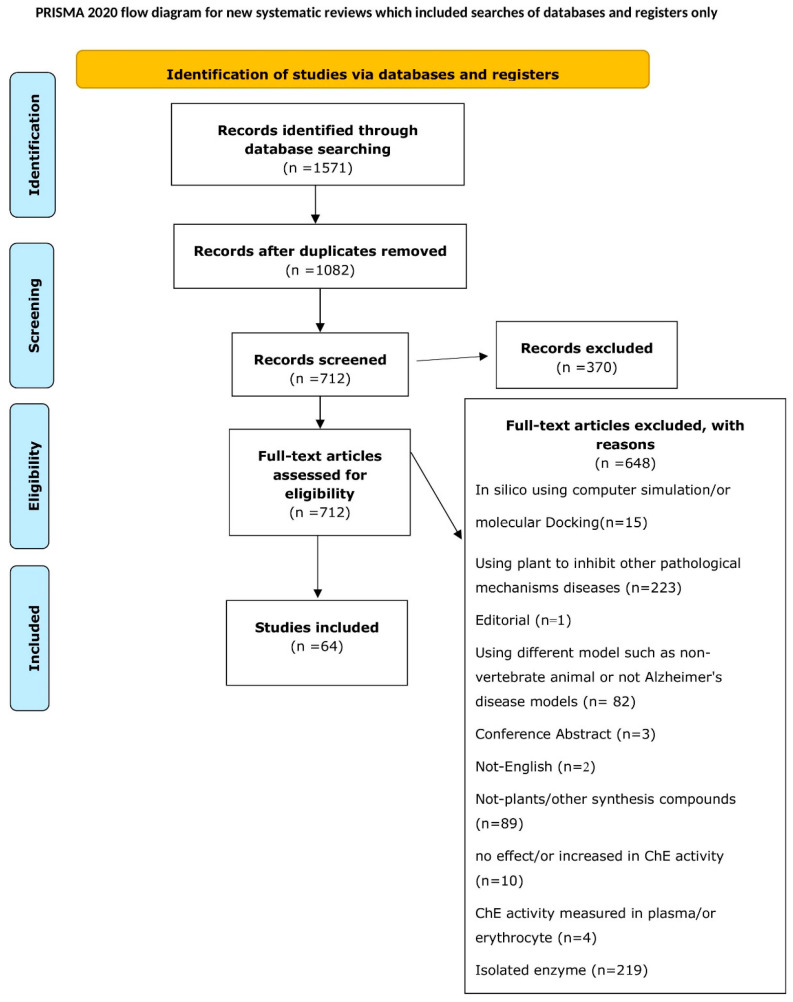
Preferred reporting items for systematic reviews and meta-analysis (PRISMA) flow chart displaying the study identification and selection process.

A total of 1571 articles were identified through the primary database search. After removing duplicates, 370 papers were excluded based on title and abstract screening, leaving 712 articles for full-text evaluation. Of these, 648 did not meet the predefined eligibility criteria and were excluded for the following reasons: in silico studies using computer simulation or molecular docking (n = 15), studies using plants to inhibit other pathological mechanisms (n = 223), editorials (n = 1), studies using non-vertebrate animals or models unrelated to Alzheimer’s disease (n = 82), conference abstracts (n = 3), non-English articles (n = 2), studies not involving plants and using other synthetic compounds (n = 89), studies showing no effect or an increase in ChE activity (n = 10), studies measuring ChE activity in plasma or erythrocytes (n = 4), and studies involving isolated enzymes (n = 219). As a result, 64 articles met the inclusion criteria. A flow chart, following the PRISMA protocol, was created to illustrate the study selection process (Figure 2).

All of the studies utilized in vivo animal models of Alzheimer’s disease (AD), with various compounds employed to induce the condition. These included scopolamine hydrobromide (SCO) (n = 39), aluminum chloride (AlCl_3_) (n = 9), streptozotocin (STZ) (n = 4), amyloid beta (Aβ) (n = 6), trimethyltin (TMT) (n = 2), transgenic dementia models (n = 2), hydrogen peroxide (H_2_O_2_) (n = 1), and 3-nitropropionic acid (3-NPA) (n = 1) (Table 1). Moreover, the results of this systematic review indicated that various animal species were used in the included studies, including the following: Swiss albino mice (n = 14), Balb/C albino mice (n = 3), C57BL/6J mice (n = 3), C57BL/6N mice (n = 3), ICR mice (n = 6), Kunming mice (n = 2), double transgenic mice (n = 2), mice (n = 4), zebrafish (n = 1), albino Wistar rats (n = 16), and albino Sprague Dawley rats (n = 12).

### 3.1. Neuroprotective Effects of Plant Extracts in Mitigating Scopolamine (SCO)-Induced Memory Impairment Through AChE Inhibition

Several studies have demonstrated the potential of various plant extracts in mitigating SCO-induced memory impairment by inhibiting AChE activity. For instance, the administration of ethyl acetate extract of *Ferula ammoniacum* and hydro-methanolic extract of *Elaeagnus umbellata* significantly reduced AChE and BChE activity in the cortex and hippocampus, enhancing memory performance [25,26].

Similarly, oral administration (o.p.) of *Salicornia europaea* ethanol extract (SE-EE), which contains Acanthoside B (Aca.B), at doses of 10–20 mg/kg significantly decreased AChE levels in both the hippocampus and cortical regions compared to the scopolamine-treated group (*p* < 0.05) [27]. Other plant extracts such as *Drynaria quercifolia*, administered intraperitoneally, significantly inhibited AChE activity in scopolamine-induced memory-impaired mice in a dose-dependent manner (*p* < 0.0001) [28]. In addition, the administration of *Vernonia amygdalina* alkaloid-rich extract (BLAE) at doses of 100 and 200 mg/kg showed a protective effect by reducing AChE and BChE activity, which were elevated by scopolamine [29].

Extracts from *Amygdalus spinosissima* roots, *Peganum harmala*, *Trianthema portulastrum*, *Centella asiatica*, and other plants, including *Citrus reticulata* and *Cudrania tricuspidata*, have also shown promising results in reducing AChE activity and improving cognitive function in SCO-treated mice [30,31,32,33,34,35]. Moreover, *Sapindus laurifolia* and walnut oil significantly reduced oxidative stress and AChE levels (*p* < 0.001) [36,37].

Furthermore, *Bacopa monnieri* and *Olax subscorpioidea* have demonstrated significant inhibition of AChE activity and oxidative stress alongside improvements in memory and neuronal integrity [38,39]. Other studies on plant extracts like *Nepeta nuda, Indigofera sessiliflora, Dendropanax morbifera*, *Pinus densiflora*, *Dichrocephala integrifolia*, *Alnus rugosa*, *Salvia moorcroftiana*, and *Sanguisorba minor* have similarly reported reduced AChE activity and enhanced cognitive function in SCO-induced memory impairment models [40,41,42,43,44,45,46,47].

The ethanolic extract of *Stachys sieboldii* (250 and 500 mg/kg) improved memory, reduced AChE activity, increased acetylcholine (ACh) and antioxidant enzyme catalase (CAT) levels, and enhanced neuroplasticity in rats and mice, suggesting neuroprotective effects [48].

Further investigations into extracts from *Ocimum basilicum*, *Lavandula stoechas*, *Schisandra chinensis,* and *Ginkgo biloba* have highlighted their neuroprotective effects, with significant reductions in AChE activity (*p* < 0.001) and improvements in memory and oxidative stress markers [49,50,51,52,53].

Studies on the methanolic extracts of *Bergenia ciliata*, *Cnestis ferruginea*, and *Emblica officinalis* also reported significant reductions in AChE activity and improvements in cognitive functions [54,55,56]. Moreover, chlorogenic acid extract demonstrated neuroprotective effects by reducing AChE activity, oxidative stress, and memory impairment, while wild ginseng also improved learning and reduced cholinergic loss and AChE activity in the hippocampus [57,58]. Other notable plant extracts, such as *Gelsemium sempervirens*, *Diospyros lotus*, and *Lavandula angustifolia*, have demonstrated improved cognitive performance and significant reductions in AChE activity, further supporting their potential neuroprotective benefits [59,60,61]. Finally, Nine-Herb Decoction, containing various plant extracts, and *Prunella vulgaris* aqueous extract have also exhibited protective effects against SCO-induced memory impairments by inhibiting AChE activity [62,63]. These findings emphasized the potential of natural plant extracts in treating memory impairment and cholinergic dysfunction in neurodegenerative diseases (NDDs), including Alzheimer’s, by inhibiting cholinesterase activity.

### 3.2. Cholinesterase Inhibition by Plant Extracts in Aluminum Chloride (AlCl_3_)-Induced Alzheimer’s Models

Treatment with various plant extracts has demonstrated potential neuroprotective effects against AlCl_3_-induced neurotoxicity. For instance, oral administration of three *Canna indica* extracts (derived from aerial parts, roots, and a combination of both) at 200 mg/kg significantly reduced AChE levels in the cortex and hippocampus (*p* < 0.001) and BChE levels in the cortex (*p* < 0.0001) [64]. Similarly, *Echinacea purpurea* extracts (250 mg/kg, aqueous and alcoholic) improved cognitive behavior, reduced oxidative stress, lowered amyloid plaque density, and inhibit AChE in rats, suggesting its neuroprotective properties [65]. Asiatic acid (AA) from *Centella asiatica* (75 mg/kg) enhanced antioxidant activity and reduced AChE activity, oxidative stress, and brain damage, especially in the cortex and hippocampus of AlCl_3_-exposed rats (*p* ≤ 0.05 and *p* ≤ 0.01, respectively) [66].

Moreover, genistein and chickpea ethanol extracts (10 mg/kg) mitigated AlCl_3_-induced neurotoxicity by reducing AChE activity, inflammation, and oxidative stress [67]. *Opuntia ficusindica* extracts (100 mg/kg) improved learning and memory, reduced AChE activity, and restored neurotransmitter levels in Sprague Dawley rats [68]. Orange peel extract (100–200 mg/kg) also significantly decreased AChE activity and mRNA expression and protected brain tissue by decreasing oxidative stress in AlCl_3_-treated rats [69]. *Malva neglecta* methanolic extract (200–600 mg/kg) and *Tecoma stans* extract (200–400 mg/kg) both improved cognition, reduced AChE activity, and alleviated oxidative stress in AlCl_3_-treated mice [70,71]. Additionally, intraperitoneal (i.p.) administration of crude ethyl alcohol extract of *Mentha longifolia* at a dose of 400 mg/kg significantly inhibited the AChE increase caused by AlCl_3_ exposure (50.42% compared to the vehicle group), with a 36.60% inhibition observed when the extract was administered to the animals [72].

### 3.3. Cholinesterase-Inhibitory Properties of Plant Extracts in Streptozotocin (STZ)-Induced Alzheimer’s Disease Models

The results from this systematic review found that four different plant extracts have demonstrated cognitive-enhancing and ChE-inhibiting properties in AD models. *Evolvulus alsinoides*, studied at doses of 100–500 mg/kg, showed antioxidant, cholinergic (AChE, and BChE), and rho kinase inhibitory effects, leading to dose-dependent cognitive improvement and reduced oxidative stress in STZ -treated rats [73]. *Clitoria ternatea* extract (100–500 mg/kg) significantly decreased AChE and BuChE activities in the cerebral cortex and hippocampus of STZ-exposed rats [74]. The hydromethanol extract (HME) of *Allium cepa*, administered at doses of 42, 84, and 168 mg/kg, reversed the increased AChE activity in STZ-treated mice, effectively restoring cognitive function [75]. Similarly, *Bergenia ciliata* methanolic extract (125–500 mg/kg) normalized AChE and BuChE activities in STZ-treated rats, highlighting its potential as a therapeutic agent for AD [76].

### 3.4. Neuroprotective Effects of Plant Extracts and Essential Oils in Amyloid Beta (Aβ)-Induced Alzheimer’s Disease Models

Various plant extracts and essential oils have demonstrated neuroprotective effects against Alzheimer’s-like symptoms by modulating AChE activity and enhancing cognitive function. *Chamaecyparis obtusa* essential oil (1 mL/cage via inhalation) in rats exhibited cognitive improvement, decreased AChE activity, and prevented neuronal apoptosis after Aβ-induced damage [77]. *Bacopa monnieri* extract (40–80 mg/kg) reduced Alzheimer’s-like symptoms in Aβ_42_-injected rats by improving cognitive function, preserving hippocampal neurons, modulating the GSK-3β/Wnt/β-catenin pathway, and significantly decreasing AChE activity at 80 mg/kg [78]. *Pinus densiflora* extract (15–30 mg/kg) improved cognitive function and reduced AChE activity, while increasing antioxidant levels, reducing neuroinflammation, and enhancing mitochondrial activity in Aβ-treated ICR mice [79]. *Cistanche tubulosa* aqueous extract (100–200 mg/kg) improved cognitive function, reduced amyloid deposition, and reversed cholinergic and dopaminergic dysfunction, with the 200 mg/kg dose significantly decreasing cortical AChE activity in Aβ-infused rats [80]. Blueberry leaf extract (ethyl acetate fraction, 5–20 mg/kg) alleviated Aβ-induced memory impairment by inhibiting AChE activity and oxidative stress in Aβ-treated mice [81]. Additionally, essential oils from *Pistacia khinjuk* leaves (PKEO) and *Allium sativum* cloves (ASEO) showed strong AChE and BChE inhibitory effects in rats, suggesting their potential as treatments for Alzheimer’s [82].

### 3.5. Neuroprotective Effects of Poncirus Trifoliata and Black Soybean Extracts in Trimethyltin (TMT)-Induced Memory Impairment Models

In TMT-induced memory impairment models, *Poncirus trifoliata* extract (400–1200 mg/kg) improved learning and memory and reduced AChE activity, highlighting its potential for NDDs [83]. Similarly, black soybean nonanthocyanin extracts (5–20 mg/kg) enhanced learning and memory, reduced oxidative stress, and inhibited AChE activity, with the 20 mg/kg dose showing significant AChE reduction in TMT-treated mice [84].

### 3.6. Neuroprotective Effects of β-Sitosterol and Valeriana Officinalis Extracts in Transgenic Alzheimer’s Disease Models

In a double-transgenic Alzheimer’s model, β-sitosterol (10 mg/kg) from *Polygonum hydropiper* enhanced memory and motor coordination and reduced AChE and BChE activity, indicating its neuroprotective potential [85]. *Valeriana officinalis* extracts, particularly volvalerenic acid K, inhibited AChE activity in a dose-dependent manner and improved learning and memory in APPswe/PS1E9 double-transgenic mice [86].

### 3.7. Neuroprotective Effects in Hydrogen Peroxide (H_2_O_2_) and 3-Nitropropionic Acid (3-NPA)-Treated Models

In H_2_O_2_-treated rats, oral administration of hydrophobic fractions from *Thymus algeriensis* (180 mg/kg/day) significantly reduced elevated ROS and AChE levels, alleviating neuronal degeneration and restoring AChE activity to near control levels (*p* < 0.05) [87]. In the 3-NPA-treated model, oral administration of *Pedalium murex* Linn leaf extract (200–400 mg/kg) improved memory, learning, and motor coordination, increased antioxidant enzymes, and decreased AChE levels in rats with Alzheimer’s-like symptoms [88].

**Table 1:** The table presents the plants and plant extracts that exhibited cholinesterase-inhibitory effects.

Reference	Study Type and Animal Species	Type of Enzyme (AChE/BChE)	Scientific Name of the Plant Type of Extracts	Dosage of the Extracts Duration/Route of Treatment	Outcomes
Adeniyi et al. (2024) [39]	In vivo Male Swiss albino mice	AChE	*Olax subscoprioidea* Oliv. (*Olacaceae*) leaves Methanol extract	25, 50, and 100 mg/kg, 14 days, p.o.	MEOS pre-treatment significantly decreased AChE activity when compared to SCO-treated group (*p* < 0.05).
Akbaba et al. (2021) [40]	In vivo Female Wistar rats	AChE	*Nepeta nuda* ssp. nuda essential oil (EO)	1% or 3%, 21 days of daily inhalation (15 min per day)	Inhalation of essential oil in SCO-treated rats demonstrated potential AChE inhibitory activity (*p* < 0.0005).
Alam et al. (2023) [60]	In vivo Balb/C albino mice	AChE	The roots of *Diospyros lotus* Diospyrin (a naphthoquinone derivative)	5, 10, and 15 mg/kg	A significant decrease in AChE enzyme activity was observed with diospyrin at 10 mg/kg (*p* < 0.05) and 15 mg/kg (*p* < 0.01) in the frontal cortex, compared to the SCO group. In the hippocampus, treatment with diospyrin at 10 mg/kg resulted in a significant decrease (*p* < 0.01) in AChE enzyme activity, while a more substantial reduction (*p* < 0.001) was observed at 15 mg/kg, compared to the SCO group.
Arora et al. (2018) [33]	In vivo Male Wistar rats	AChE	*Centella asiatica* (CA) Compared different extracts of CA: CA extract, enriched for triterpenes (CAE-EF) and depleted/freed of triterpenes (CAE-FF), with methanolic extract (CAE) and scopolamine injection on day 15	100 mg/kg, 15 days, p.o.	The increase in AChE activity in the cerebral cortex caused by SCO was also considerably inhibited by CAE (*p* < 0.01). In the rat brain’s cerebral cortex and hippocampus, CAE-EF and CAE-FF also inhibited the rise in AChE activity brought on by SCO.
Arora et al. (2021) [38]	In vivo Male Wistar rats	AChE	*Bacopa monnieri* (BM) Methanolic extract of BM (BME), bacosides-enriched fraction (BME-EF), and bacosides-free fraction (BME-FF)	100 mg/kg, 14 days, p.o.	The elevated AChE induced by SCO was also prevented with BME and BME-EF pre-treatment and differences found were significant at *p* < 0.01 and *p* < 0.05, respectively.
Bandaru et al. (2020) [36]	In vivo Albino Wistar rats	AChE	*Sapindus laurifolia* Methanolic extract (MESL)	MESL fraction A (catechin) 50 and 100 mg/kg, MESL crude extract 250 and 500 mg/kg, 7 days, i.p.	Treated animals significantly showed a decrease in AChE levels (*p* < 0.001) compared to SCO-treated animals.
Barai et al. (2018) [54]	In vivo Albino Wistar male rats	AChE BuChE	*Bergenia ciliata* (Haw) Sternb. methanolic extracts (BM)	125, 250, and 500 mg/kg, 14 days, p.o.	A concentration-dependent decrease in AChE activity was observed, with the 250 mg/kg and 500 mg/kg doses effectively mitigating the spike in brain AChE activity, resulting in a significant reduction (*p* < 0.001) compared to the SCO-injected group. The BuchE activity in rats pre-treated with 250 and 500mg/kg BM showed significantly attenuated escalation (*p* < 0.05) as compared to the SCO-injected group.
Brinza et al. (2021) [47]	In vivo Zebrafish (*Danio rerio*)	AChE	*Alnus rugosa* leaves Flavonoid-isolated baicalein 5,6-dimethyl ether	1, 3, and 5 µg/L, 10 days, immersion in water for zebrafish	The 100 µM SCO-treated zebrafish that received baicalein 5,6-dimethyl ether showed a significant dose-depending decrease in AChE activity (*p* < 0.01 and *p* < 0.001) compared to SCO-alone-treated fish.
Das et al. (2002) [52]	In vivo Male Swiss mice	AChE	*Ginkgo biloba* Standardized extracts	15, 30, and 60 mg/kg daily, 7 days, postoperatively, daily as aqueous suspension	AChE-specific activity was significantly lowered in the detergent-soluble fraction (DS) of the 30 and 60 mg/kg *G. biloba*-treated dementia groups (*p* < 0.001, indicating a significant difference from the SCO-treated dementia group).
Deng et al. (2019) [31]	In vivo Male C57BL/6J mice	AChE	Aerial parts of *Peganum harmala* Linn Deoxyvasicine (DVAS) is one of the chief active ingredients in *P. Harmala*	5, 15, and 45 mg/kg, 7 days, p.o.	DVAS treatment significantly reduced AChE and ache levels in hippocampus and cortex in mice treated with scopolamine, with varying doses affecting the brain’s function. Treatment with DVAS significantly attenuated the increase in the AChE level (*p* <0.05, *p* < 0.01, *p* < 0.001) in hippocampus and cortex in SCO-treated mice.
Ferdous et al. (2024) [28]	In vivo Adult Swiss albino mice	AChE	*Drynaria quercifolia* The crude methanol extract of rhizome: ethyl acetate (DEF), chloroform (DCF)	DCF (200, 100, and 50 mg/kg) and DEF (400, 200, and 100 mg/kg), 12–15 successive days, i.p.	Treatment with DCF and DEF resulted in a significant, dose-dependent reduction in AChE activity (*p* < 0.0001) in the brains of SCO-induced memory-impaired mice.
Golechha et al. (2012) [55]	In vivo Swiss albino mice	AChE	Fruit of *Emblica offcinalis* Hydroalcoholic extract	150, 300, 450, 600 mg/kg, 7 days, i.p.	The EO extract at doses of 150 mg/kg (*p* < 0.01), 300 mg/kg, 450 mg/kg, and 600 mg/kg (*p* < 0.001) significantly and dose-dependently reduced the SCO-induced elevation of AChE levels in the mice brain.
Haider et al. (2021) [41]	In vivo Male Sprague Dawley rats	AChE	The whole plant of *Indigofera sessiliflora* (IS.CR) Aqueous-methanol crude extract (IS.CR)	100, 200, and 300 mg/kg, 48 days, p.o..	Increased levels of AChE induced via SCO were prominently reduced in the brains of animals, with IS.CR at doses of 200 (*p* < 0.01) and 300 mg/ kg (*p* < 0.0001) as compared to the SCO-treated group.
Hosseini et al. (2022) [46]	In vivo Male Wistar rats.	AChE	*Sanguisorba minor* Hydro-ethanolic extract	50, 100, and 200 mg/kg, 21 days, i.p.	Animals treated with different doses of the extract (50, 100, and 200 mg/kg, *p* < 0.001) exhibited a reduction in AChE activity in the hippocampus, compared to the SCO group. The highest dose of the extract (200 mg/kg) significantly reduced AChE activity in the cortex compared to the SCO group (*p* < 0.05).
Ishola et al. (2013) [56]	In vivo Male Swiss albino mice	AChE	*Cnestis ferruginea* Vahl ex (CF) methanol root extract (CF) and its active constituent amentoflavone (CF-2)	CF (100 and 200 mg/kg/bw, p.o.) and CF-2 (12.5, 25 mg/kg/bw, p.o.), 3 days	The AChE activity was significantly decreased in CF-treated groups (100 and 200 mg/kg) when compared to the SCO group (*p* < 0.01). CF-2 (12.5 and 25 mg/kg) significantly reduced AChE activity at both doses (*p* < 0.01) in comparison to the SCO group.
Jee et al. (2020) [35]	In vivo Male mice	AChE	*Cudrania tricuspidata* Fruit-methanol-(CTFE)	125, 250, and 500 mg/kg/day, 28 days, p.o.	The SCO + 500 mg/kg/day CTFE group showed a decrease in AChE activity in hippocampal tissue compared to the SCO group.
Karthivashan et al. (2019) [27]	In vivo Male C57BL/6N mice strain	AChE	*Salicornia europaea* SE ethanol extract (SE-EE), isolation and identification of Acanthoside B (Aca.B) from SE-EE	Aca.B 10–20 mg/kg, 7 days, p.o.	Aca.B-treated groups showed a significant decrease in AChE levels in both hippocampus and cortical regions compared to SCO-treated groups (*p* < 0.05).
Kim et al. (2023) [42]	In vivo C57BL/6N male mice	AChE	The dried aerial parts of *Dendropanax morbifera* Water extract from leaves and stems (DMLS)	125, 250, and 375 mg/kg, 3 weeks, p.o.	Treatment with all DMLS concentrations significantly reduced AChE activity compared to the SCO-treated group (*p* < 0.01).
Kouémou et al. (2017) [44]	In vivo Swiss mice model	AChE	*Dichrocephala integrifolia* leaves Decoction of leaves	35, 87.5, 175, and 350 mg/kg, 10 days, p.o.	The activity on AChE induced via SCO was significantly reduced by *D. integrifolia* at the dose of 87.5 mg/kg (*p* < 0.0001).
Kwon et al. (2010) [57]	In vivo Male ICR mice	AChE	Chlorogenic acid (CGA) The extract type is a purified compound rather than a crude plant extract	3, 6, and 9 mg/kg, 7 days, p.o	CGA administration (3 or 6 mg/kg) significantly inhibited AChE activity in the hippocampus by 21.67% and 35.06% compared with the SCO-treated group (*p* < 0.05 and *p* < 0.01, respectively) but did not significantly inhibit AChE activity in the frontal cortex at these concentrations. CGA administration (9 mg/kg) also significantly inhibited AChE activities in the hippocampus and frontal cortex by 56.49% and 35.52% (*p* < 0.001 and *p* < 0.05, respectively).
Lee et al. (2015) [43]	In vivo C57BL/6N male mice	AChE	*Pinus densiflora* (Japanese red pine) 30% ethanolic extract of pine needle (PNE)	25, 50, and 100 mg/kg, 7 days, p.o.	Pre-treatment with PNE completely inhibited the hyper-activation of AChE compared with the SCO-injection group (*p* < 0.001 for all groups (25, 50, and 100 mg/kg for PNE)).
Lee et al. (2010) [58]	In vivo Male Sprague Dawley rats	AChE	*Panax ginseng* Methanol extract of WG roots (adventitious root culture of *Panax ginseng*)	Wild ginseng (WG): 50, 100, and 200 mg/kg, 7 days, i.p. Cultivated ginseng (CG): 500 mg/kg, 7 days, i.p.	AChE activity was reduced in the WG groups, indicating a potential improvement in the cholinergic system. The density of AChE-reactive neurons in the hippocampus was significantly lower in the WG100 + DEM group (*p* < 0.05) and the WG200 + DEM group (*p* < 0.01) compared to the SCO-induced group, especially in the CA1 region.
Liao et al. (2020) [37]	In vivo Kunming mice	AChE	Walnut oil	10 mL/kg, 8 weeks, p.o.	Walnut oil significantly inhibited the SCO-induced increase in AChE activity in brain tissue (*p* < 0.05 versus the SCOP-treated group).
Mushtaq et al. (2021) [50]	In vivo Swiss albino mice	AChE	*Lavandula stoechas* L. (aerial parts) Aqueous extract (AfL.s) isolated through column chromatography	9 and 18 mg/kg, 7 days, p.o.	The AChE level was significantly reduced in comparison to SCO-treated animals, with the group treated with AfL.s (18 mg/kg, p.o.) showing the greatest inhibition of AChE among all groups (*p* < 0.001).
Nazir et al. (2021) [25]	In vivo Swiss male albino mice	AChE BChE	Aerial parts extract/fractions of *Ferula ammoniacum* (*Dorema ammoniacum*) Ethyl acetate (Fa.etac)	50, 100, and 200 mg/kg, 8 days, p.o.	In the Fa.etac + SCOP-treated groups, a significant decrease (*p* < 0.01, *p* < 0.001) in %AChE and %BChE activities was observed in the frontal cortex and hippocampus tissues, with levels lower than those in the SCO-treated group.
Nazir et al. (2020) [26]	In vivo Swiss male albino mice	AChE BChE	*Elaeagnus umbellata* Hydro-methanolic extract (Met.Ext) Isolated compound 1 (chlorogenic acid) CGA Chloroform (CHF.Ext)	Compound 1 (10 and 30mg/kg, 8 days, i.p.), CHF extract (200 mg/kg, 8 days, i.p.)	The results showed that the % AChE and BChE activity in the cortex and hippocampus tissues of the CGA- and CHF-treated groups were significantly lower (*p* < 0.001, *p* < 0.01) compared to the SCO-treated group.
Oboh et al. (2022) [29]	In vivo Albino rats	AChE BChE	*Vernonia amygdalina* Bitter leaf alkaloid-rich extract (BLAE)	100, 200 mg/kg, 7–14 days	AChE and BChE activity were elevated by SCO; however, these effects were mitigated by BLAE pre-treatment (*p* < 0.05).
Palit et al. (2015) [59]	In vivo Male Swiss albino mice	AChE	*Gelsemium sempervirens* L. Reconstituted hydro-alcoholic mother tincture	1 mg/kg, 14 days, p.o.	Gelsemium significantly reduced AChE activity in comparison to SCO-treated mice (*p* < 0.01). Gelsemium pre-treatment in SCO-injected mice profoundly decreased mRNA expression level of AChE in comparison to only SCO-treated dementia mice.
Pruthi et al. (2021) [34]	In vivo Mice	AChE	*Citrus reticulata* Methanol extract (ME) of *Citrus reticulata* var. Kinnow leaves (CR) Leaf extracts (EAFs) of ethyl acetate fraction	ME (200 and 400 mg/kg) EAF fraction (25 and 50 mg/kg), 7 days, p.o.	By decreasing brain AChE activity, ME and EAF both enhanced the cognitive function that SCO caused in mice (*p* < 0.05 vs. SCO).
Qu et al. (2017) [63]	In vivo Male Wistar rats	AChE	*Prunella vulgaris* L. Aqueous extract (EtOAc-APV)	100 mg/kg, 3 days, p.o.	In rats with SCOP-induced brain dementia, oral administration of EtOAc-APV significantly inhibited the increase in AChE activity (*p* < 0.05).
Ravichandran et al. (2018) [48]	In vivo Sprague Dawley rats and ICR mice	AChE	*Stachys sieboldii* (SS) Ethanolic extract	250 and 500 mg/kg, 28 days, p.o.	The treatment with the extract significantly decreased the elevated levels of AChE induced via SCO to the control group by both concentrations in the hippocampus (*p* < 0.05).
Sajjadi et al. (2021) [30]	In vivo Male mice	AChE BChE	*Amygdalus spinosissima* root parts Methanolic extract	50, 100, or 150 mg/kg, 14 days, p.o.	The treatment of SCO-injected mice with the extract showed significant (*p* < 0.05) down-regulation of the AChE and BChE genes.
Singh et al. (2022) [49]	In vivo Swiss albino mice	AChE	Leaves of *Ocimum basilicum* (sweet basil) Isolation of two pure compounds, namely 5,7-dihydroxy-3′,4′,5′-trimethoxyflavone (S1) and 3-hydroxy-3′,4′,5′-trimethoxyflavone (S2)	5 and 10 mg/kg, 7 days, p.o.	The compounds isolated from OBE (S1 and S2) significantly reduced AChE activity induced in SOC-treated mice (*p* < 0.05 vs. SCO).
Singh et al. (2016) [53]	In vivo Balb/C mice	AChE	Leaves of *Ocimum basilicum* Hydro-methanol extracts	200 and 400 mg/kg, 7 days, i.p.	SCO administration significantly increased AChE activity, while OBE pre-treatment notably reduced brain AChE activity (*p* < 0.05).
Song et al. (2020) [51]	In vivo KM mice	AChE	*Schisandra chinensis* (SC) Aqueous and alcohol extracts	10 mg/kg, 18 days, p.o.	The AChE activity was increased in SCO-AD model group and decreased to a level close to the normal group by the treatment with SC extract (*p* < 0.01 vs. SCO model group).
Wahid et al. (2022) [45]	In vivo Male Balb/C mice	AChE	*Salvia moorcroftiana* Crude methanolic extract (SlMo-Crd) and fractions (hexane; SlMo-Hex, chloroform; SlMo-Chl, ethyl acetate; SlMo-Et)	SIMO-Crd (100 and 200 mg/kg) SlMo-Hex, SlMo-Chl, SlMo-Et (75 and 150 mg/kg), 28 days, p.o.	All treatments resulted in a decrease in AChE levels in the hippocampus and frontal cortex, which were elevated by SCO treatment (*p* < 0.05–*p* < 0.001 vs. SCO-treated (amnesic) group).
Weon et al. (2016) [62]	In vivo ICR mice	AChE	Nine-Herb Decoction with Notopterygium, also known as Chianghuo Combination (GT), consists of nine crude extracts obtained from the following: *Ostericum koreanum*, *Saposhnikovia divaricata*, *Cnidium officinale*, *Angelica dahurica*, *Atractylodes japonica*, *Scutellaria baicalensis*, *Rehmannia glutinosa*, *Asiasarum sieboldi*, and *Glycyrrhiza uralensis* Nine-Herb Decoction (fermented GT)	50, 100, and 200 mg/kg, 4 days, p.o.	Fermented GT treatment significantly decreased AChE activity in the hippocampus of the SCO-treated group by 24.15% and 31.64% at doses of 100 mg/kg and 200 mg/kg, respectively (*p* < 0.05 compared to the SCO-treated group).
Xu et al. (2016) [61]	In vivo C57BL/6J mice	AChE	*Lavandula angustifolia* The type of extract used in this study is lavender essential oil (LO), which is an aromatic liquid isolated from *Lavandula angustifolia*	50 and 100 mg/kg/d, 10 days, p.o.	The LO (100 mg/kg) decreased the AChE activity in SCO-treated mice (*p* < 0.05).
Yadav et al. (2019) [32]	In vivo Male Swiss albino mice	AChE	*Trianthema portulastrum* (TP) Butanol fraction of TP hydro-ethanolic extract (BFTP)	200, 400, and 600 mg/kg/day, 21 days, p.o.	BFTP (600 mg/kg, *p* < 0.01) demonstrated an ameliorative effect on SCO-induced elevation of AChE activity, while lower doses of BFTP (400 and 200 mg/kg, *p* < 0.05) also improved the cholinergic deficit caused by SCO.
Ibrahim et al. (2016) [72]	In vivo Male Sprague Dawley rats	AChE	*Mentha longifolia* Crude extract with ethyl alcohol	400 mg/kg, 60 days, i.p.	AChE was significantly increased by AlCl_3_ (50.42% compared to vehicle group) but inhibited by 36.60% when crude extract was given to the animal.
Saleem et al. (2021) [70]	In vivo Wistar albino rats	AChE	*Malva neglecta* Methanolic extract (MNME)	200, 400, and 600 mg/kg, 21 days, p.o.	The AChE levels in the MNME-treated groups significantly decreased in a dose-dependent manner, while those in the AlCl_3_-treated groups rose (*p* < 0.001).
Abd El-Aziz et al. (2023) [69]	In vivo Male albino rats	AChE	Orange peel Aqueous extraction (OPE)	100 and 200 mg/kg, 6 weeks, p.o.	Rats treated with AlCl_3_ (*p* < 0.05) and given 100 or 200 OPE showed significantly lower levels of AChE activity and mRNA in their brain tissue.
El-Hawary et al. (2020) [68]	In vivo Sprague Dawley rats	AChE	*Opuntia ficusindica* (cladode, peel, and fruits) Methanol extracts from cladode, peel, and fruit pulp The polysaccharides of both fruits and cladodes	100 mg/kg, 6 weeks, p.o.	All the studied extracts (cladode, peel, fruits, polysaccharides extract) showed a significant decrease in AChE levels counteracting the effect of AlCl_3_ (*p* < 0.05 versus AlCl_3_ group).
Gouthami et al. (2020) [71]	In vivo Swiss albino mice	AChE	*Tecoma stans* Methanolic extract of leaves	200 or 400 mg/kg, 42 days, p.o.	AlCl_3_-treated mice showed increased brain AChE activity, while METS administration significantly decreased AChE levels compared to the control group.
Mohamed et al. (2023) [65]	In vivo Male Wistar rats	AChE	*Echinacea purpurea* flowers Aqueous and alcoholic extracts	250 mg/kg, 60 days, p.o.	Both extracts significantly inhibit AChEs in vivo compared to the AlCl_3_-treated group (*p* < 0.0001).
Ojha et al. (2022) [64]	In vivo Male Wistar rats	AChE BChE	The aerial parts and root of *Canna indica* L. Methanolic extract of aerial parts (CIA) and hydroalcoholic extract of roots (CIR)	200 mg/kg, 21 days, p.o.	CIA, CIR, and CIA + CIR extracts significantly decreased the level of AChE in the cortex and hippocampus compared to the AlCl_3_ group (*p* < 0.001). CIA, CIR, and CIA + CIR significantly decreased the level of BChE in the cortex when compared to the AlCl_3_ group (*p* < 0.0001). Only CIA and CIA + CIR significantly decreased the level of BChE in the hippocampus as compared to the AlCl_3_ group (*p* < 0.0001).
Suryavanshi et al. (2022) [66]	In vivo Male Wistar rats	AChE	*Centella asiatica* Asiatic acid (AA)	75 mg/kg, 8 weeks, p.o.	Co-administration of AA effectively declined the AChE activity in the cortex (*p* ≤ 0.05) and hippocampus (*p* ≤ 0.01) of the brain as compared to AlCl_3_-intoxicated rats.
Wahby et al. (2017) [67]	In vivo Male albino Sprague Dawley rats	AChE	*Cicer arietinum* (chickpea) The isoflavones were extracted from chickpea (*Cicer arietinum*) seeds Genistein (Gen) (lisoflavone) and chickpea ethanol extract (CPE)	10 mg/kg, 6 weeks, p.o.	Co-administration of either Gen or CPE with AlCl_3_ significantly decreased the activity of AChE, restoring it to the control value (*p* < 0.01).
Mehla et al. (2013) [74]	In vivo Male Wistar rats	AChE BuChE	*Clitoria ternatea* Hydroalcoholic extracts of whole plant	100, 300, and 500 mg/kg, 7 days before the ICV-STZ injection and continued for 28 days after injection, p.o.	A significant decrease in AChE activity was observed in the cerebral cortex and hippocampus following extract administration, compared to the STZ group (*p* < 0.05, *p* < 0.01, and *p* < 0.001). BuChE activity in the cerebral cortex and hippocampus was significantly reduced following extract administration (*p* < 0.01 and *p* < 0.001), compared to the STZ group.
Kaur et al. (2020) [75]	In vivo Swiss albino mice	AChE	*Allium cepa* Hydromethanol extract (HME)—ethylacetate fraction (EAF) of HME	EAF (42, 84, and 168 mg/kg, 9–22 days, p.o.)	ICV STZ-treated mice showed a significant increase in the brain AChE activity compared with the control group and this was efficiently reversed in EAF-treated mice (*p* < 0.05 vs. STZ control group)
Barai et al. (2018) [76]	In vivo Male albino Wistar rats	AChE BChE	*Bergenia ciliata* Rhizomes—methanolic extracts (BM)	125, 250, and 500 mg/kg, 28 days, p.o.	BM restored the AChE activity to normal levels and showed a dose-dependent decrease in the increased AChE activity caused by STZ (*p* < 0.001). BM significantly reduced the STZ-induced increase in BuChE activity and returned it to normal levels at all concentrations (*p* < 0.05–*p* < 0.001).
Mehla et al. (2012) [73]	In vivo Male Wistar rats	AChE BChE	*Evolvulus alsinoides* Aqueous and hydro-alcoholic	100, 300, and 500 mg/kg, 35 days, p.o.	A significant decrease in AChE activity was observed with hydro-alcoholic extract of *E. alsinoides* at doses of 100 mg/kg (*p* < 0.05), 300 mg/kg (*p* < 0.001), and 500 mg/kg (*p* < 0.001) in cerebral cortex and hippocampus as compared to STZ group. A significant decrease in BuChE activity was observed in cerebral cortex with hydro-alcoholic extract of *E. alsinoides* at doses of 100 mg/kg (*p* < 0.05), 300 mg/kg (*p* < 0.01), and 500 mg/kg (*p* < 0.001) as compared to STZ group. In hippocampus, a significant decrease in BuChE activity was observed with hydro-alcoholic extract of *E. alsinoides* at 300 mg/kg (*p* < 0.05) and 500 mg/kg (*p* < 0.001) as compared to STZ group.
Bae et al. (2012) [77]	In vivo Male Sprague Dawley rats	AChE	*Chamaecyparis obtusa* essential oil (EOCO)	1 mL/cage for two consecutive hours for 30 days before injection of Aβ into the hippocampus and 7 days after injection, inhalation	Inhalation of EOCO significantly reduced AChE activity in rats injected with Aβ-induced cognitive impairment (*p* < 0.05).
Ghajarbeygi et al. (2019) [82]	In vivo Male Wistar rats	AChE BChE	*Pistacia khinjuk* and *Allium sativum* essential oils Essential oils (EOs) from Iranian *Pistacia khinjuk* leaves (PKEO) and *Allium sativum* cloves (ASEO)	1, 2, and 3% wt/wt, 3 weeks, p.o.	Oral administration of PKEO and ASEO resulted in significant inhibition of AChE and BChE in rats injected with Aβ_25–35_ peptide (*p* < 0.05).
Go et al. (2022) [79]	In vivo Male ICR mice	AChE	*Pinus densiflora* (Korean red pine) Water extract of Korean red pine bark (KRPBE)	15 and 30 mg/kg, p.o.	Both concentrations showed decrease in AChE activity compared to the Aβ group (*p* < 0.05). The AChE protein expression level of the P30 group was partially down-regulated compared to the Aβ group (*p* < 0.05).
Jeong et al. (2013) [81]	In vivo ICR mice	AChE	Blueberry (*Vaccinium angustifolium*) Leaf extracts by ethyl acetate (EtOAc) fraction	5, 10, and 20 mg/kg, 3 weeks, p.o.	The Aβ-treated group showed an increase in AChE activity, whereas the pre-treatment of the EtOAc fraction (5, 10, 15 mg/kg) effectively inhibited AChE in the brains of mice after Aβ_25–35_ exposure (*p* < 0.05).
Sushma et al. (2024) [78]	In vivo Male Wistar rats	AChE	*Bacopa monnieri* (BM) Ethanolic extract	40 and 80 mg kg−1, 28 days, p.o.	BM 80 mg kg−1 significantly decreased the AChE activity that was elevated by Aβ_42_ injection in the hippocampal tissue of rat brains, compared to the Aβ_42_-injected group (*p* < 0.05).
Wu et al. (2014) [80]	In vivo Male Sprague Dawley rats	AChE	*Cistanche tubulosa* (CT) Aqueous extract	100 and 200 mg/kg, 14 days, p.o.	CT extract (200 mg/kg) only reversed the increase in cortical AChE activity in Aβ_1–42_-infused rats (*p* < 0.01 for CT).
Jeong et al. (2014) [84]	In vivo ICR mice	AChE	Soybeans (*Glycine max* (L.) Merr) Extracts from black soybean (*Glycine max*), specifically freeze-dried nonanthocyanins from black soybean	5,10, and 20 mg/kg, 3 weeks, oral—in drinking water	The pre-treatment groups with nonanthocyanins relatively inhibited AChE activity in the brains of mice. AChE activity in the (20 mg/kg)-treated group was significantly reduced compared to the TMT-treated group (*p* < 0.05 vs. TMT-treated group).
Kim et al. (2009) [83]	In vivo Mice	AChE	*Poncirus trifoliata* Ethanol extract, further fractionated to isolate methoxsalen	400, 800, and 1200 mg/kg, 3 weeks, mixed in the diet	All sample groups exhibited significant inhibition of AChE activity induced by the TMT injection (*p* < 0.05 vs. TMT group).
Ayaz et al. (2017) [85]	In vivo (APPSwFlLon, PSEN1∗M146L∗L286V) Double-transgenic mice JAX^®^Strain	AChE BChE.	*Polygonum hydropiper* Isolated β-sitosterol	10 mg/kg, 5 days, i.p.	In the frontal cortex, β-sitosterol caused a significant decrease in AChE and BChE levels (*p* < 0.05, *p* < 0.01). β-sitosterol was able to significantly decrease BChE levels (*p* < 0.05) in the hippocampus, compared to saline-treated transgenic animals.
Chen et al. (2016) [86]	In vivo C57BL/6J male mice were used as the normal control group with Class SPF APPswe/PS1E9 double-transgenic dementia mice	AChE	The root of *Valeriana officinalis* var. *latiofolia* Solvent extracts, fractionation, and purification Compound 4 (volvalerenic acid K)	0.65, 1.30, and 2.60 mg·kg^−l^·day^−1^, 90 days, p.o.	Administration of Compound 4 (volvalerenic acid K) resulted in a significant, concentration-dependent decrease in AChE activity in the brain of the transgenic dementia mouse model (*p* < 0.01).
Guesmi et al. (2018) [87]	In vivo Male Sprague Dawley rats	AChE	The aerial parts of *Thymus algeriensis* Hydrophobic fractions	180 mg/kg/day, 15 days, p.o	The extract significantly reduced the elevated AChE levels in the brains of H_2_O_2_-treated rats, restoring AChE activity to levels comparable to the normal control group (*p* < 0.05).
Velayutham et al. (2023) [88]	In vivo Sprague Dawley rats	AChE	*Pedalium murex* Linn Ethanolic extract of *Pedalium murex* Linn leaf (EEPML)	200 and 400 mg/kg, 14 days, p.o.	The elevated AChE levels induced by 3-NPA administration in rats were significantly reduced following EEPML treatment (*p* < 0.001).

Abbreviations: AChE: acetylcholinesterase; AlCl_3_: aluminum chloride; Aβ: amyloid beta; BChE: butyrylcholinesterase; H_2_O_2_: hydrogen peroxide; i.p.: intraperitoneal injection; ICV: intracerebroventricularly; p.o.: oral administration; 3-NPA: 3-nitropropionic acid; SOC: scopolamine hydrobromide; STZ: streptozotocin; TMT: trimethyltin.

## 4. Discussion

The systematic review of the literature presented substantial evidence supporting the neuroprotective effects of various plant extracts in alleviating memory impairment associated with Alzheimer’s disease (AD) through the inhibition of acetylcholinesterase (AChE) and/or butyrylcholinesterase (BChE) activities. AChE is a pivotal enzyme responsible for the hydrolysis of acetylcholine (ACh), a neurotransmitter crucial for cognitive function. The decline in ACh levels due to increased AChE activity is a hallmark of AD, contributing to cognitive decline and memory impairment [16]. In addition to AChE, BChE plays a significant role in the pathophysiology of AD. While AChE is primarily responsible for terminating cholinergic neurotransmission, BChE has been shown to hydrolyze ACh as well, albeit with lower catalytic efficiency [89]. Notably, during the progression of AD, BChE activity can increase significantly, potentially contributing to the aggregation of amyloid beta-peptides and the formation of senile plaques, which are characteristic of the disease [90,91]. Therefore, the identification of natural compounds that can inhibit both AChE and BChE represents a promising therapeutic strategy for AD, as it may help restore cholinergic function and mitigate the neurodegenerative processes associated with this condition [92,93].

Numerous studies included in this review demonstrated that various plant extracts significantly reduced AChE and BChE activities and improved cognitive performance in animal models of AD. For instance, the ethyl acetate extract of *Ferula ammoniacum* and the hydro-methanolic extract of *Elaeagnus umbellata* were shown to effectively decrease both AChE and BChE activity in the cortex and hippocampus, leading to enhanced memory performance in scopolamine (SCO)-treated mice [25,26]. Similarly, administration of extracts from *Vernonia amygdalina*, *Amygdalus spinosissima*, and *Bergenia ciliata* demonstrated protective effects against SCO-induced cognitive impairment by significantly reducing both AChE and BChE activity in treated animals [29,30,54]. The cholinergic hypothesis of AD suggested that impaired cholinergic neurotransmission contributes to cognitive decline. Research indicated that in patients with advanced AD, AChE levels can decline significantly while BChE levels may increase, suggesting a compensatory mechanism where BChE attempts to hydrolyze ACh in the absence of sufficient AChE activity [92]. Additionally, increased BChE activity, especially in the hippocampus and temporal cortex, may exacerbate this cognitive decline by promoting amyloid-beta aggregation and plaque formation, which are key features of AD [94,95]. Therefore, the ability of these plant-derived compounds to inhibit both AChE and BChE indicates a promising therapeutic strategy for restoring cholinergic function and alleviating cognitive impairments associated with AD [96,97].

Moreover, the review highlighted the multifaceted mechanisms through which these plant extracts exerted their neuroprotective effects. Many of the extracts not only inhibited ChE activity but also exhibited antioxidant properties, thereby reducing oxidative stress—a key factor in the pathogenesis of NDDs [98,99]. For instance, extracts from *Bacopa monnieri* and *Olax subscorpioidea* demonstrated significant reductions in oxidative stress alongside AChE inhibition, suggesting that these compounds may protect neuronal integrity while enhancing cognitive function [38,39]. The interplay between cholinergic modulation and antioxidant activity underscores the potential of these natural products as comprehensive therapeutic agents against AD.

In addition to SCO-induced models, the review also examined the effects of plant extracts in aluminum chloride (AlCl_3_)-induced neurotoxicity. Extracts from *Canna indica* and *Echinacea purpurea* significantly reduced AChE levels and improved cognitive behavior in AlCl_3_-treated rats, indicating that these plants may offer protective effects against neurotoxic agents commonly associated with AD [64,65]. The ability of *Centella asiatica* to enhance antioxidant activity while reducing AChE activity further emphasizes the potential of these extracts in combating neurodegenerative processes [66]. The review also highlights the efficacy of plant extracts in models of AD induced by streptozotocin (STZ) and amyloid beta (Aβ). For example, *Evolvulus alsinoides* and *Clitoria ternatea* exhibited significant AChE inhibition and cognitive improvement in STZ-treated rats, indicating their potential as therapeutic agents for AD [73,74]. Furthermore, essential oils and extracts from *Chamaecyparis obtusa* and *Bacopa monnieri* had shown promise in ameliorating Aβ-induced cognitive deficits, further supporting the role of phytochemicals in neuroprotection [77,78].

This study supported recent review studies, highlighting the potential of plant extracts and their isolated active compounds with anti-cholinesterase properties in advancing drug discovery for AD [100,101,102]. The diverse mechanisms through which these plant extracts exert their effects—ranging from ChE inhibition to antioxidant activity—suggested that they may serve as valuable adjuncts or alternatives to conventional pharmacological treatments for AD [103,104]. As the search for effective treatments continues, the integration of traditional herbal medicine with modern pharmacological approaches may pave the way for novel strategies in managing Alzheimer’s disease and other neurodegenerative disorders.

## 5. Conclusions

This study identified plant species with bioactive compounds that inhibit AChE and BuChE, offering potential therapeutic benefits for AD. Additionally, many of the extracts demonstrated not only ChE inhibition but also antioxidants, anti-inflammatory properties, as well as enhanced learning and memory, suggesting they could serve as effective alternatives or supplements to conventional AD treatments.

## Data Availability

Data supporting the results are available on request from the first author (M.N.A.). The data are not publicly available due to confidentiality agreements.

## Supplementary Materials

The following supporting information can be downloaded at https://www.mdpi.com/article/10.3390/brainsci15020215/s1: Table S1: PRISMA checklist.
